# Inhibition of Epithelial-Mesenchymal Transition Maintains Stemness in Human Amniotic Epithelial Cells

**DOI:** 10.1007/s12015-022-10420-1

**Published:** 2022-08-06

**Authors:** Chika Takano, Masafumi Horie, Isamu Taiko, Quang Duy Trinh, Kazunori Kanemaru, Shihoko Komine-Aizawa, Satoshi Hayakawa, Toshio Miki

**Affiliations:** 1 Division of Microbiology, Department of Pathology and Microbiology, Nihon University School of Medicine, Tokyo, Japan; 2 Department of Pediatrics and Child Health, Nihon University School of Medicine, Tokyo, Japan; 3 Department of Molecular and Cellular Pathology, Graduate School of Medical Sciences, Kanazawa University, Kanazawa, Japan; 4 Department of Physiology, Nihon University School of Medicine, Tokyo, Japan

**Keywords:** Human amniotic epithelial cells, Epithelial-mesenchymal transition, Stemness, TGF-β pathway, Comprehensive transcriptome analysis

## Abstract

Human amniotic epithelial cells (hAECs), which are a type of placental stem cell, express stem cell marker genes and are capable of differentiating into all three germ layers under appropriate culture conditions. hAECs are known to undergo TGF-β-dependent epithelial-mesenchymal transition (EMT); however, the impact of EMT on the stemness or differentiation of hAECs has not yet been determined. Here, we first confirmed that hAECs undergo EMT immediately after starting primary culture. Comprehensive transcriptome analysis using RNA-seq revealed that inhibition of TGF-β-dependent EMT maintained the expression of stemness-related genes, including *NANOG* and *POU5F1*, in hAECs. Moreover, the maintenance of stemness did not affect the nontumorigenic characteristics of hAECs. We showed for the first time that TGF-β-dependent EMT negatively affected the stemness of hAECs, providing novel insight into cellular processes of placental stem cells.

**Graphical abstract:**

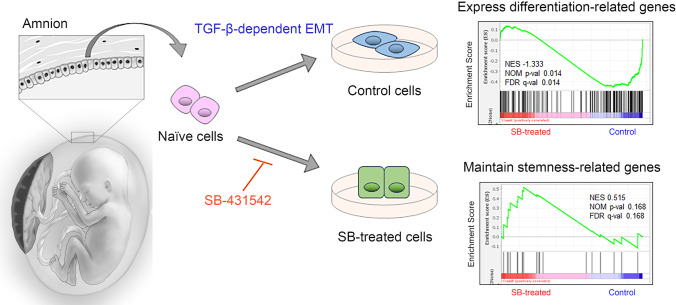

**Supplementary Information:**

The online version contains supplementary material available at 10.1007/s12015-022-10420-1.

## Introduction

Human amniotic epithelial cells (hAECs) have attracted attention as a new source of regenerative therapy based on a number of unique characteristics [1–4]. First, as hAECs develop from epiblasts by the eighth day after fertilization, they partially maintain the plasticity of pregastrulation embryo cells [1]. Evidence has been provided by the expression levels of NANOG, octamer-4 (Oct-4), and sex determining region Y-box 2 (SOX-2), which are known genes related to self-renewal and pluripotency. Moreover, hAECs express stem cell surface markers, such as stage-specific embryonic antigen-3 (SSEA-3) and SSEA-4 and tumor rejection antigen 1–60 (TRA1-60) and TRA1-81, which are known to be expressed on human embryonic stem cells (ESCs). Under appropriate culture conditions, hAECs can differentiate into cells of all three germ layers: ectoderm (neuronal cells), mesoderm (cardiomyocytes), and endoderm lineage cells (liver, pancreas cells) [1, 2]. Second, the placenta is an immune privilege organ. Due to the low expression of major histocompatibility complex (MHC) class 1 and the lack of MHC class 2, hAECs have a low immunogenic profile [5]. Third, hAECs possess wide-ranging immunomodulatory effects. They secrete soluble HLA-G and other factors that suppress proinflammatory cytokines and inhibit the chemotactic activity of neutrophils and macrophages [6]. Fourth, and more importantly, hAECs have no tumorigenicity, as proven by transplantation studies in humans [7]. Based on this evidence, several preclinical studies have proven the therapeutic potential of hAECs [8, 9]. For cell transplantation therapies, determining how the stemness of hAECs is regulated is essential.

One of the biological processes that might affect stemness and differentiation is the transforming growth factor-β (TGF-β)-dependent epithelial-mesenchymal transition (EMT) [10]. It is known that EMT is involved in development, wound healing, and cancer metastasis [11]. In the process of development, stem cells differentiate into various tissues via EMT. Hamidi et al. demonstrated that EMT during gastrulation coincides with the loss of pluripotency using chicken epiblasts, mammalian ESCs and induced pluripotent stem cells (iPSCs) [12]. In the derivation of iPSCs, the mesenchymal-epithelial transition (MET) was recognized as an efficient process, and TGF-β-dependent EMT must be circumvented to stabilize the pluripotent state of iPSCs [13, 14]. On the other hand, TGF-β signaling is essential for maintaining the pluripotency of human ESCs [15], and the inhibition of TGF-β signaling results in differentiation into the neuroectoderm [16]. Thus, it has been shown that the TGF-β-dependent EMT can affect both stemness maintenance and differentiation depending on the cell type and the biological situation.

It has been reported that hAECs undergo EMT in response to autocrine stimulation with TGF-β [17]. However, the impact of the TGF-β-dependent EMT on the stemness or differentiation of hAECs has not yet been determined. Based on several findings in previous EMT studies, we hypothesized that the TGF-β-dependent EMT would affect the stemness of hAECs. In this study, we first confirmed the presence of the TGF-β-dependent EMT in cultured hAECs by using a selective inhibitor of the TGF-β pathway. Then, we performed comprehensive transcriptome analysis using RNA-seq to identify the impact of the EMT on the cell transcriptome. Lastly, we verified the nontumorigenic characteristics of hAECs after EMT inhibition.

## Materials and Methods

### Isolation and Cultivation Of Human Amniotic Epithelial Cells (hAECs)

Under the approval of the institutional review board (IRB) (No. 29–4-2) of Nihon University School of Medicine, hAECs were isolated from the placentae of 10 patients. The criteria for placental donation were as follows: scheduled cesarean section for term deliveries, no indication for pathologic examination, and no evidence for infectious diseases such as hepatitis B or HIV. Informed consent and written agreements were obtained prior to delivery. To avoid contamination by normal vaginal flora, donor placentas were collected at the time of cesarean section under sterile conditions.

The protocol for hAEC isolation was described previously [18]. In brief, the amnion layer was mechanically separated from the chorion layer of the placenta and washed several times with phosphate-buffered saline without calcium and magnesium (PBS). To dissociate the hAECs, the amniotic membrane was incubated at 37 °C for 40 min with 0.05% trypsin twice. Each trypsin digest was inactivated with Hank’s balanced salt solution (HBSS) supplemented with 10% fetal bovine serum (FBS). The cells were pooled, washed with HBSS/FBS, filtered with a 100 µm cell strainer (Corning Inc., NY, USA) and counted. The viability of the hAECs was determined by exclusion of trypan blue dye and counted with a hemocytometer. The cells were cryopreserved using a controlled rate freezer with GMP-grade Stem cell banker (Thermo Fisher Scientific, MA, USA) at 10 million cells per vial.

The standard medium for hAEC cultivation was high glucose Dulbecco’s modified Eagle’s medium (DMEM) supplemented with 10% FBS (Nichirei Corporation, Japan), 1% penicillin/streptomycin, 1% l-glutamine, 1% nonessential amino acid, and 55 μM 2-mercaptoethanol (Thermo Fisher Scientific, MA, USA). Recombinant human epidermal growth factor, which is often added to hAEC culture medium [18], was not added to the medium in this study in order to evaluate the specific effect of the TGF-β pathway inhibitor. hAECs were seeded at 1.25 × 10^5^ cells/cm^2^ to achieve an adequate cell density. hAECs were cultured and harvested on day 1, day 4 and day 7 to analyze the sequential changes in EMT-related gene expression levels. After optimizing the concentration of SB-431542 (Cayman Chemical Company, MI, USA), hAECs were cultured for 7 days in the presence or absence of SB-431542, which was added immediately after thawing the cryopreserved cells to avoid initiation of the TGF-β-dependent EMT. Phase-contrast photographs were taken by a DMi8 microscope (Leica, Germany).

### mRNA Expression Analysis

Total RNA was extracted from hAECs using TRIzol reagent (Thermo Fisher Scientific, MA, USA) and RNA clean and concentrator (Zymo Research, CA, USA). The amount of RNA was measured by Nanodrop, and reverse transcription was performed with an iScript Supermix kit (Bio–Rad Laboratories, CA, USA) from 1 µg of RNA. RT–qPCR was performed using SYBR SuperMix (Bio-Rad Laboratories, CA, USA) equipped with a QuantStudio3 Real-Time PCR System (Applied Biosystems, MA, USA) according to the manufacturer’s instructions. Gene expression was normalized to the mRNA expression of an internal control (*PPIA*) by the delta-delta Ct method. The primers used for gene amplification are shown in Table S1.

### Immunocytochemistry

hAECs cultured on cover glasses were fixed for 15 min at room temperature (RT) with 4% formaldehyde/PBS. After washing with PBS, permeabilization was performed with 0.3% Triton X-100 in PBST for 15 min. The samples were incubated with blocking buffer consisting of PBS supplemented with 3% bovine serum albumin and Tween 20 for 30 min. Subsequently, they were incubated with the appropriate primary antibody diluted in blocking buffer overnight and washed three times with PBS. After primary antibody incubation, the samples were incubated with the appropriate secondary antibodies diluted in blocking buffer for 30 min at RT. The primary antibodies were anti-E-cadherin (1:75, Abcam, UK) and anti-N-cadherin (1:100, Abcam, UK), and the secondary antibodies were goat anti-rabbit IgG (Alexa Fluor 488 for E-cadherin and Alexa Fluor 594 for N-cadherin, Abcam, UK) at a 1:250 concentration. Then, samples were mounted with ProLong Glass Antifade Mountant with NucBlu™ (Thermo Fisher Scientific, MA, USA) for nucleus detection and stored at 4 °C. Images were taken by an Olympus IX3 inverted fluorescence microscope (OLYMPUS, Japan).

### Western Blotting

hAECs were lysed with PROPREP (iNtRON Biotechnology, MA, USA) supplemented with Phosphatase Inhibitor Cocktail (EDTA free) (Nakarai tesque, Japan). Protein extracts were analyzed by sodium dodecyl sulfate polyacrylamide electrophoresis (SDS–PAGE; Bio-Rad Laboratories, CA, USA) followed by a Western blot assay. EzBlockChemi (ATTO, Japan) was used for blocking, and Western BLoT Immuno Booster (Takara, Japan) was used for the reaction. The primary antibodies were as follows: Smad1, Smad2/3, phospho-Smad1 (Ser463/465)/5 (Ser463/465)/9 (Ser465/467), and phospho-Smad2 (Ser465/467)/3 (Ser423/425) (Cell Signaling Technology, MA, USA) at a 1:1,000 concentration, and GAPDH (GeneTex, CA, USA) at a 1:10,000 concentration. The secondary antibodies were peroxidase AffiniPure goat anti-mouse IgG and anti-rabbit IgG (Jackson ImmunoResearch Laboratory, PA, USA) at a 1:20,000 concentration. Western blot images were developed with Western BLoT Hyper HRP Substrate (Takara, Japan) and taken with a LumiCube System (Liponics, Japan).

### RNA-seq and Bioinformatic Analysis

Total RNA was extracted from hAECs cultured for 7 days with and without SB-431542 using TRIzol reagent (Thermo Fisher Scientific, MA, USA) and RNA clean and concentrator (Zymo Research, CA, USA). Comprehensive transcriptome analysis was performed using a total of six RNA samples (SB-treated; *n* = 3, Control; *n* = 3). All samples had RNA integrity number (RIN) values ranging from 9.7 to 10 using an Agilent 2100 Bioanalyzer (Agilent Technologies, CA, USA). Since these samples passed the internal quality control test and did not show any deviation from the other samples in the multidimensional scaling analysis, they were included in further analyses. Samples were prepared for sequencing using an Illumina TruSeq RNA Sample Prep Kit (Illumina, CA, USA). Sequencing was performed by a NovaSeq 6000 system (Illumina, CA, USA) using the standard Illumina RNAseq protocol with a read length of 2 × 100 bases.

Paired-end sequencing was performed at Macrogen Japan (Tokyo, Japan), and fastq files were obtained. The raw sequencing data were evaluated by Fast QC ver.0.11.9 (Available at https://www.bioinformatics.babraham.ac.uk/projects/fastqc/), and then we confirmed their quality for use in the following analyses. We trimmed the sequences with Trimmomatic [19]. The index was made with a human transcript sequence (GRCh38.p13) obtained from GENCODE 39 (09.12.21, available at https://www.gencodegenes.org/), and pseudoalignment was preformed using kallisto ver. 0.46.1 [20]. The annotations for protein-coding genes and cDNA were made by biomaRt package ver. 2.50.1 [21] using R ver. 4.0.3. The expression analysis was performed with the tximport package ver.1.18.0 [22]. Differentially expressed genes (DEGs) were identified between two groups using the read counts of 19,944 protein-coding genes. The Wald test was performed by DESeq2 ver. 1.30.0 [23], which was selected due to the high consistency of the results [24]. Gene ontology analysis was performed by Cluster Profiler ver. 3.18.1 [25]. To determine the cell states of differentiation and stemness, we performed Gene Set Enrichment Analysis (GSEA) ver. 4.1.0 (available at https://www.gsea-msigdb.org/gsea/index.jsp) using read counts of 38,979 cDNA genes. We searched the gene sets for the word “STEMCELL” and selected the 20 genes in common with at least 2 gene sets among the 5 gene sets (BENPORATH_ES_2, BHATTACHARYA_EMBRYONIC_STEM_CELL, GOBP_STEM_CELL_PROLIFERATION, GOBP_SOMATIC_STEM_CELL_POPULATION, CONRAD_STEM_CELL) as stemness-related genes. Three gene sets, ectoderm development (GO:0,007,398), endoderm development (GO:0,007,492), and mesoderm development (GO:0,007,498), were combined as differentiation-related genes. We also performed GSEA with regionalized genes (GO: 0,003,002), which relate to pattern specification in developmental processes. The genes used for GSEA are shown in Table S2.

### FACS Analysis

Cultured hAECs with or without SB-431542 were prepared, and human dermal fibroblasts (batch no: 21TL116652, Lonza, Switzerland) were used as a negative control. The cells were suspended in 1 mL of HBSS containing 2% FBS. After supplementation with 10 µL rBC2LCN-FITC (AiLecS1-FITC, Excitation 495 nm, Emission 520 nm; FUJIFILM, Japan), the cells were incubated at room temperature for 30 min in the dark. Following washing twice with PBS, the population of FITC-labeled cells was analyzed by BD Verse flow cytometry (Becton, Dickinson and Company, NJ, USA). 7-Amino-actinomycin D (BioLegend, CA, USA) was utilized to exclude dead cells. The data were analyzed using FlowJo software ver. 10.8.1 (Becton, Dickinson and Company, NJ, USA).

### Soft Agar Colony Formation Assay

A CytoSelect™ 96-Well Cell Transformation Assay Kit (Cell Biolabs, CA, USA) was used in this study. hAECs cultured for 7 days with or without SB-431542 were plated in 1.2% soft agar in a 96-well plate at 10,000 cells per well. HeLa cells were used as a positive control, and all of the samples were cultured for 7 days. To measure anchorage-independent colony growth, the agar layers were dissolved and lysed. Ten microliters of the lysed solutions of each well were mixed with CyQuant working solution. Then, the fluorescence was read using a 485/520 nm filter set by a multimode plate reader (Infinite M Plex, Tecan Japan Co., Japan).

### Statistical Analyses

Statistical analysis was performed with JMP software ver. 14. The obtained data were compared using the Wilcoxon rank-sum test. For correcting for multiple comparisons, the Tukey–Kramer test was used. A p value < 0.05 was considered statistically significant.

## Results

### hAECs Underwent TGF-β-dependent EMT Shortly after Starting Cell Culture

**Fig. 1:**
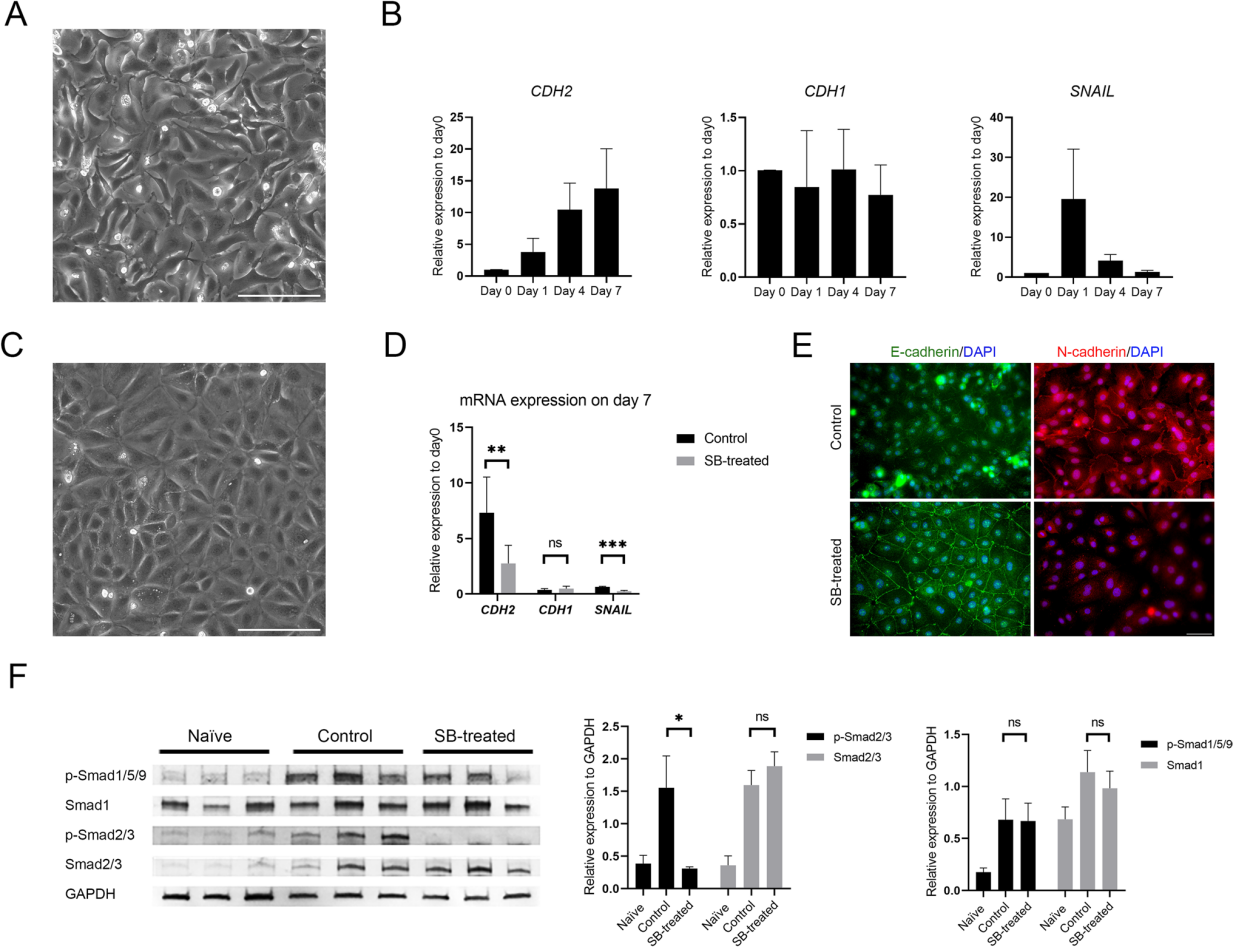
TGF-β-dependent EMT in hAECs and the effect of SB-431542 supplementation. (**A**) Phase-contrast photograph of control cultured hAECs at day 7. Scale bar: 200 μm. (**B**) RT–qPCR for the indicated genes of naïve (day 0) cells and three independent hAEC time-course experiments. The data are shown as the mean ± SEM (*n* = 6). (**C**) Phase-contrast photograph of cultured hAECs supplemented with SB-431542 at day 7. Scale bar: 200 μm. (**D**) RT–qPCR for the indicated genes in control and SB-treated cells at day 7. The data are shown as the mean ± SEM (*n* = 6). ***p < 0.001 (Wilcoxon rank-sum test). (**E**) Immunocytochemistry analysis of E-cadherin (green), N-cadherin (red) and DAPI (blue) for comparison between control and SB-treated cells at day 7. Scale bar: 50 μm. (**F**) Western blotting of phosphorylated Smad1/5/9, Smad1, phosphorylated Smad2/3, and Smad2/3 expression in the indicated groups. The expression was normalized to GAPDH and is shown as the mean ± SEM (*n* = 3). *p < 0.05 (Tukey–Kramer test)

During 7 days of cultivation, the cell morphology turned into a fibroblast-like appearance (Fig. 1A). As shown in Fig. 1B, the mRNA expression level of a typical mesenchymal marker, *CDH2*, was elevated, while that of *CDH1*, an epithelial marker, did not significantly decrease over this period. The mRNA expression of the EMT marker *SNAIL* was transiently upregulated one day after starting culture.

To confirm that the EMT of cultured hAECs is TGF-β dependent, a small molecule, SB-431542, which is a selective inhibitor of activin receptor-like kinase (ALK) 5 (the TGF-β type I receptor), was added to the culture media. The concentration of SB-431542 was optimized by dose-dependent experiments using RT–qPCR and cell viability assays. A concentration of 10 µM was most effective in suppressing *CDH2* mRNA expression in the cells on day 7 (Supple. Figure 1A). The cell viability assay also showed that this concentration was suitable (Supple. Figure 1B). Thus, 10 µM SB-431542 was used in the following experiments.

By inhibiting the TGF-β signaling pathway using SB-431542 (SB-treated), the cells maintained the typical cobble-stone-like epithelial morphology, as shown in Fig. 1C. The viability of SB-treated cells on day 7 was 99.9 ± 0.13%, which was comparable to that of control cells (99.2 ± 0.83%). The cell growth was also comparable between control and SB-treated cells (Supple. Figure 1C). Figure 1D shows that *CDH2* and *SNAIL* mRNA expression levels were suppressed in SB-treated cells on day 7 (*p* < 0.001), although *CDH1* mRNA expression was not significantly different between these cells and control cells (*p* = 0.81). According to the immunocytochemistry results, N-cadherin was localized to intracellular junctions in control cells, whereas E-cadherin was localized to intracellular junctions in SB-treated cells on day 7 (Fig. 1E).

The TGF-β superfamily contains two subfamilies, the TGF-β/Activin/Nodal subfamily and the BMP (bone morphogenetic protein)/GDF (growth and differentiation factor)/MIS (Mullerian inhibiting substance) subfamily, as defined by sequence similarity and the specific signaling pathways that they activate [26]. The signals are transduced by phosphorylation of receptor-regulated Smads (R-Smads). Smad2 and Smad3 respond to signaling by the TGF-β subfamily and Smads 1, 5, and 9 primarily by the BMP subfamily. To evaluate the possible crosstalk among the TGF-β signaling components, the protein expression level and phosphorylation of R-Smads were investigated by Western blot assay (Fig. 1F). The phosphorylation of Smad2/3 was significantly suppressed by the addition of SB-431542 (*p* < 0.05), although Smad2/3 protein expression was similar in control and SB-treated cells (*p* = 0.57). There was no significant difference in Smad1 protein expression or the phosphorylation of Smad1/5/9 between control and SB-treated cells.

### The Transcriptional Profile of SB-treated Cells was Similar to that of Naïve hAECs

**Fig. 2:**
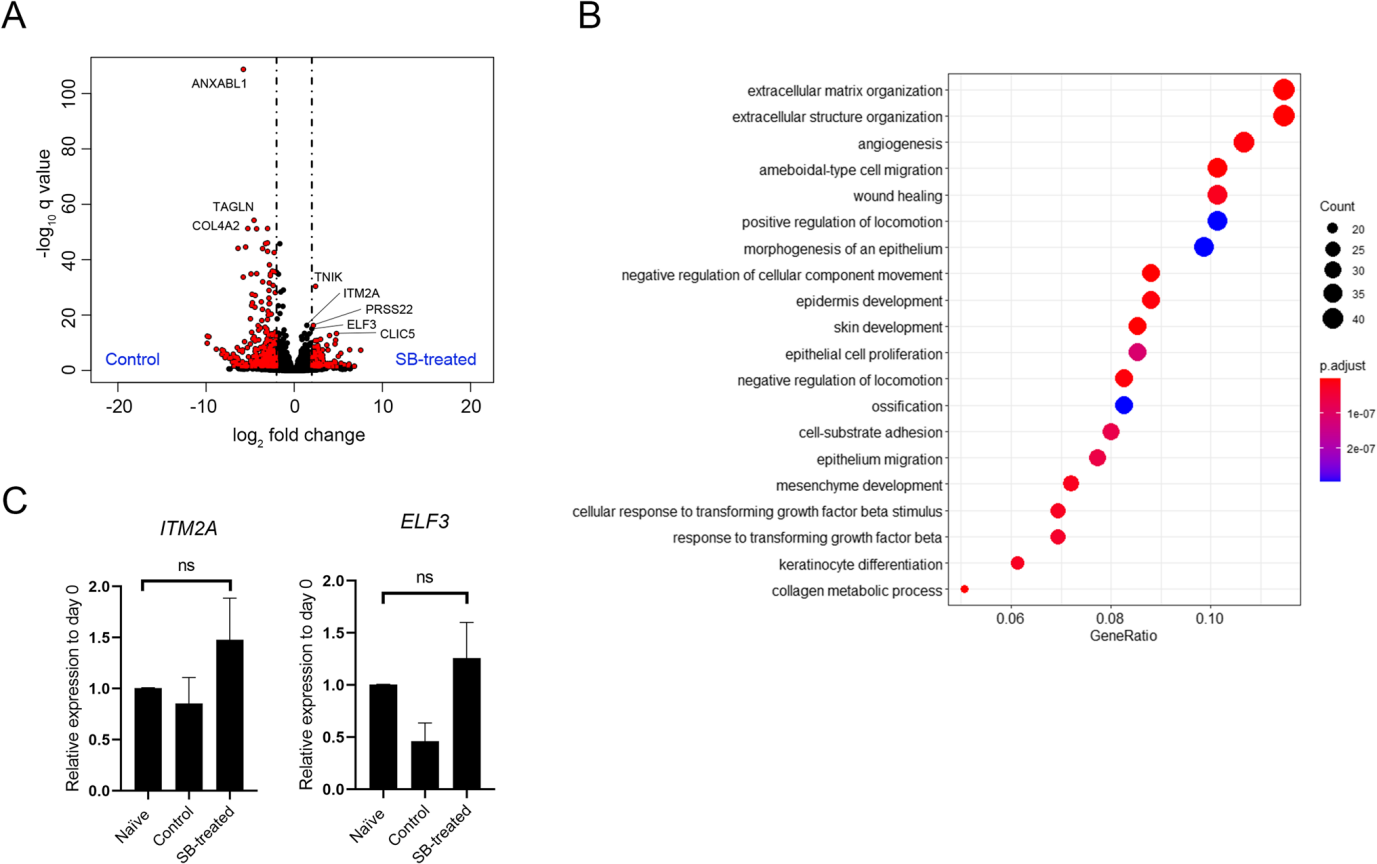
Differentially expressed genes obtained from comprehensive transcriptome analysis. (**A**) Volcano plot of DEGs obtained by the Wald test using DESeq2 for the comparison of control and SB-treated cells. The x-axis represents the log base 2 of the gene expression ratio. The y-axis represents the negative log_10_ of the adjusted q value. Red dots represent DEGs meeting the stringency cutoff; adjusted p value < 0.05 and |log_2_ fold change|> 2. (**B**) Enrichment analysis of Gene Ontology Biological Processes using Cluster Profiler. Top 20 annotations are shown. The x-axis represents the number of gene clusters in each cluster label. The color represents the adjusted p values, and the size of the spots represents the gene number. (**C**) RT–qPCR for the representative DEGs enriched in SB-treated cells. The data are shown as the mean ± SEM (*n* = 6)

Comprehensive gene expression analysis by RNA sequencing (RNA-seq) was performed using primary hAECs that were cultured for 7 days with or without SB-431542. The average read counts of the obtained raw sequencing data were 51,265,859 bp with a 50.6% GC rate. Among 19,944 protein-coding genes, 1,427 differentially expressed genes (DEGs) were identified between the two groups using the Wald test (adjusted p value < 0.05), as shown in a volcano plot (Fig. 2A). A total of 853 DEGs were upregulated in the control group. Gene ontology analysis (Fig. 2B) showed that the DEGs were enriched in extracellular matrix organization (GO:0,030,198), extracellular structure organization (GO:0,043,062), and other ontologies related to epithelial and cell movements. Among the 1,427 DEGs, 574 were enriched in SB-treated cells. We selected the top 10 DEGs and performed validation RT–qPCR to compare the gene expression levels among naïve (day 0) cells, control cells, and SB-treated cells. Nine of the investigated gene expression level differences in the RNA-seq data were verified with RT–qPCR. Two genes, namely, *ITM2A* and *ELF3,* which are related to cell differentiation, are presented in Fig. 2C. The expression levels of both genes in SB-treated cells were the same as those in naïve (day 0) cells (*ITM2A*; *p* = 0.47, *ELF3*; *p* = 0.70).

### The Stemness of hAECs was Maintained by Inhibiting TGF-β-dependent EMT

**Fig. 3:**
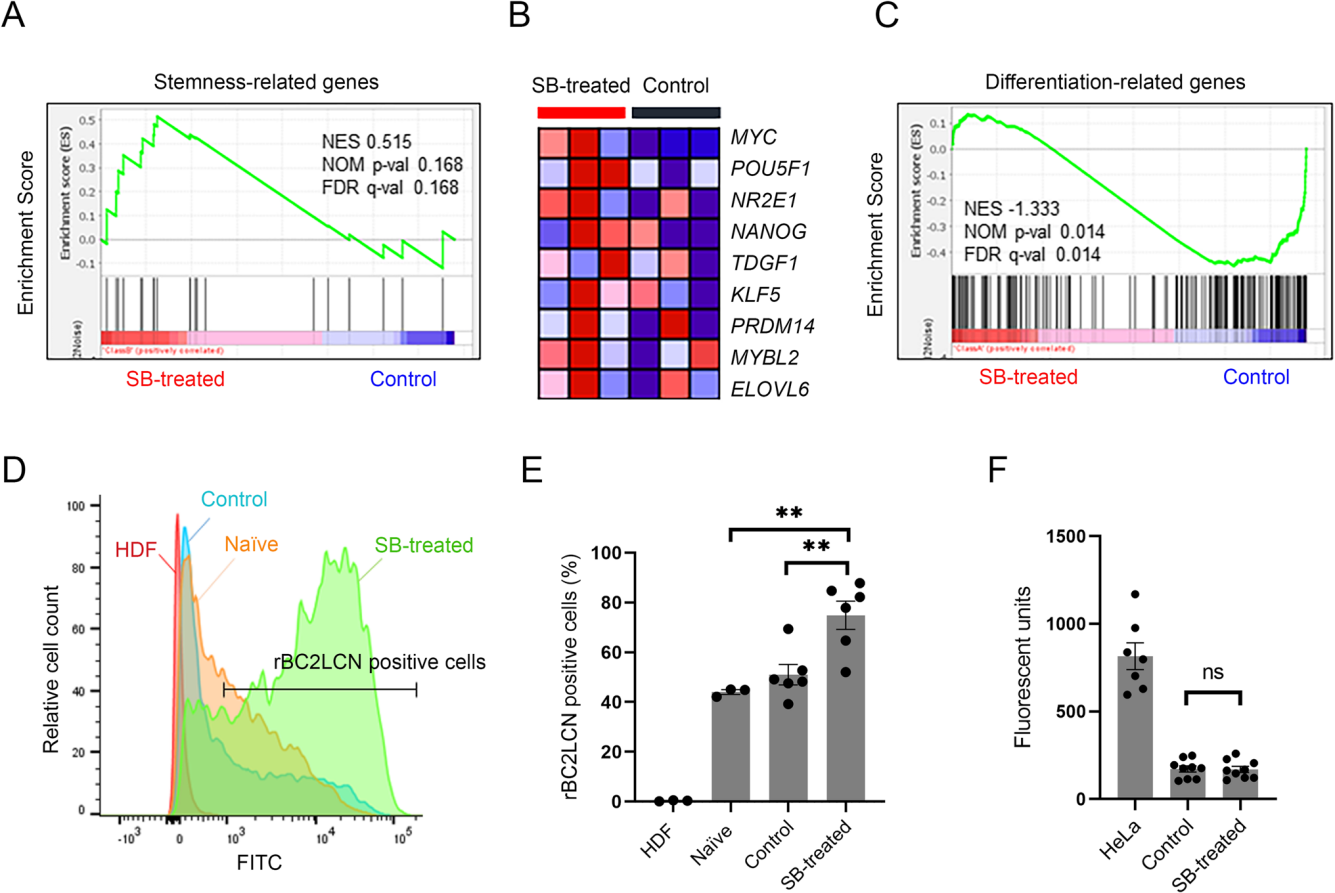
Stemness maintenance by inhibiting TGF-β-dependent EMT. (**A**) Gene set enrichment analysis (GSEA) using the gene set associated with stemness for the comparison of control (*n* = 3) and SB-treated cells (*n* = 3). (**B**) Heatmap of the leading edge subset of stemness-related genes for control and SB-treated cells. (**C**) GSEA using the gene set associated with differentiation. (**D**) Histogram of rBC2LCN-FITC-positive cells among the indicated groups (representative data) obtained by flow cytometry analysis. (**E**) Bar graph for the percentage of rBC2LCN-FITC-positive cells among the indicated groups. The data are shown as the mean ± SEM (*n* = 3). **p < 0.01 (Tukey–Kramer test). (**F**) Soft agar colony forming assay. Bar graph of fluorescent units representing anchorage-independent growth of HeLa cells, control cells, and SB-treated cells. HeLa cells were used as the positive control. The data are shown as the mean ± SEM (*n* = 3)

Using the read counts of 38,979 cDNA genes, we performed Gene Set Enrichment Analysis (GSEA). Stemness-related genes were enriched in SB-treated cells (Fig. 3A). Figure 3B shows the leading edge genes enriched in SB-treated cells, which included typical pluripotent markers, such as *POU5F1* and *NANOG*. In contrast, differentiation-related genes were significantly enriched in control cells (Fig. 3C). Regionalized genes (GO:0,003,002), which relate to pattern specification in developmental processes, were also significantly enriched in control cells (Supple. Figure 2A). When divided according to germ layer, endoderm- and mesoderm-related genes were relatively enriched in control cells, whereas ectoderm-related genes were not biased between control and SB-treated cells (Supple. Figure 2B). As shown in supplemental Fig. 3A, immunocytochemistry revealed that NANOG was localized to cell nuclei. The fluorescence intensity of SB-treated cells was significantly higher than that of control cells (*p* < 0.001) (Supple. Figure 3B).

The stemness of SB-treated cells was also investigated using FITC-labeled rBC2LCN, a lectin probe that binds specifically to the cell surface of human pluripotent stem cells [27]. Compared to human dermal fibroblasts, the population of FITC-labeled cells was markedly increased in hAECs (*p* < 0.001). The FITC-positive ratio was significantly higher in SB-treated cells than in control cells (*p* < 0.01) (Fig. 3D, and E).

Lastly, we investigated the tumorigenicity of SB-treated cells using a soft agar colony formation assay. As shown in Fig. 3F, no colony formation was observed in either SB-treated or control hAECs, indicating that stemness maintenance by SB-431542 treatment does not affect the nontumorigenic characteristics.

## Discussion

We investigated for the first time the relationship between TGF-β-dependent EMT and the stemness of hAECs using comprehensive transcriptome analysis. Our data suggest that the inhibition of TGF-β-dependent EMT maintained the stemness of hAECs. In consideration of the clinical application of hAECs, a certain culture period is required to evaluate cell viability and quality prior to cell transplantation [8, 9]. In addition, cell cryopreservation is also required to provide sufficient cell numbers and for off-the-shelf transplantation strategies. Therefore, this study focused on the use of cryopreserved hAECs after 7 days of cultivation.

hAECs underwent EMT immediately after starting cell culture. In recent years, a number of researchers have investigated the EMT during organogenesis or cancer cell metastasis [11, 28–30]. This reversible process largely depends on the cell type and environmental factors contributing to tissue repair, organ fibrosis, or carcinoma progression. Since Alcaraz et al. demonstrated that EMT-related genes were upregulated with high passage numbers in cultured hAECs [17], we first investigated mRNA expression using cells after 1, 4 and 7 days of cultivation to clarify when the EMT was initiated. The expression levels of the EMT marker genes *CDH2* and *SNAIL* were elevated overnight (Fig. 1B). These data suggest that the EMT of hAECs was initiated immediately after starting in vitro cultivation on the plastic dishes. Alcaraz et al. also reported that the EMT of hAECs was caused by the autocrine production of TGF-β, and the addition of a pathway-specific inhibitor or TGF-β neutralizing antibody prevented the EMT [17]. Therefore, in this study, SB-431542 was supplemented at the same time as cell culture was started to inhibit TGF-β-dependent EMT.

Interestingly, GSEA showed that inhibiting TGF-β-dependent EMT using SB-431542 maintained stemness-related gene expression in hAECs (Fig. 3A). To confirm the difference in NANOG protein expression between control and SB-treated cells, immunocytochemical staining of NANOG was performed (Supple. Figure 3A). The significant difference in the immunofluorescence intensity of SB-treated cells compared with that of control cells (Supple. Figure 3B) partially supported our findings. In contrast, the differentiation-related genes and regionalized genes were significantly enriched in the control cells (Fig. 3C, Supple. Figure 2A). The data suggested that hAECs could differentiate into the three germ layers (Supple. Figure 2B). These data indicated that the impact of the EMT on hAECs was similar to that of EMT-associated pluripotency loss in mammalian pluripotent stem cell models reported by Hamidi et al. [12], who concluded that the epiblast EMT followed by a partial MET was associated with complete pluripotency loss. Since hAECs are developmentally derived from epiblasts before gastrulation, we assumed that some primary hAECs still possessed characteristics similar to those of epiblasts. Thus, the EMT in culture led hAECs to differentiate. This result also supports our previous finding that hAECs can differentiate into all three germ layers [1].

The next question was whether inhibition of the Smad2/3 pathway suppressed the differentiation of all hAECs or enhanced stem cell marker gene expression in some of the hAECs. Therefore, we conducted additional experiments to elucidate the stem cell population in the SB-treated cells by flow cytometry assay using rBC2LCN. rBC2LCN is a lectin that specifically binds to the Fucα1-2Galβ1-3 motif, which was highly expressed on pluripotent stem cells [31]. Our data showed that rBC2LCN-positive hAECs were dominant in the SB-treated group (Fig. 3D, E), indicating that ALK5 inhibition increased the number of stem cell marker-positive cells and their expression levels in each cell. However, a technical limitation must be considered: although the percentage of rBC2LCN-positive naïve (day 0) cells was lower than that of SB-treated cells, this might be due to the transient loss of surface glycans. Further studies are required to elucidate the impact of trypsinization and cryopreservation on the expression of the Fucα1-2Galβ1-3 motif on hAECs.

Recently, rBC2LCN was used to remove undifferentiated iPSCs, which might possess tumorigenicity, prior to clinical application [27]. One of the promising advantages of hAECs is a lack of tumorigenicity. Thus, we performed a colony formation assay in vitro to confirm that the increase in rBC2LCN expression does not indicate that the hAECs acquire tumorigenicity. hAECs did not form any colonies in either the control or SB-treated groups (Fig. 3F). Therefore, stemness maintenance using SB-431542 treatment of hAECs is suitable for consideration in clinical application.

Stem cell-based therapies have become a subject of interest as novel strategies for various diseases. For several targets, including congenital metabolic diseases, whose phenotype could be dramatically improved if the 5% missing enzyme activity from functional hepatocytes derived from stem cells could be obtained, the therapeutic potential of human ESCs and iPSCs has been widely discussed. However, there are some limitations, including ethical issues, a risk of tumorigenicity, and difficulty in the mass adjustment of cells [32]. hAECs could overcome these limitations and may be a promising source of cells for transplantation therapy [3, 33]. This study provides an important step in understanding the regulation of stemness and differentiation of hAECs for clinical translational research.

## Conclusion

We reported here for the first time that the inhibition of the TGF-β-dependent EMT maintained the stemness of hAECs. This finding provides novel insight into the cellular processes and regulation of primary hAECs. The safety characteristics of these cells, including a lack of tumorigenicity, were also preserved regardless of modification in primary culture. Further analysis of Smad-independent TGF-β family signaling pathways or of crosstalk with other pathways, including the Wnt signaling pathway, is needed to identify the precise mechanism underlying the maintenance of hAEC stemness.

## Supplementary Information

ESM 1 (DOCX 30.2 kb)

Figure S1 (PNG 43 kb)

High resolution image (TIF 13978 kb)

Figure S2 (PNG 1302 kb)

High resolution image (TIF 35969 kb)

Figure S3 (PNG 263 kb)

High resolution image (TIF 25716 kb)

## Data Availability

The data described in this article will be shared upon reasonable request to the corresponding author.
