# Supplementary material for: Inhibition of Epithelial-Mesenchymal Transition Maintains Stemness in Human Amniotic Epithelial Cells
Source: Stem Cell Rev Rep. 2022 Aug 6;18(8):3083–91. doi: 10.1007/s12015-022-10420-1 (PMC9622541; doi:10.1007/s12015-022-10420-1)
Supplement: Supplementary file 1 — (DOCX 30.2 kb) [file 12015_2022_10420_MOESM1_ESM.docx]

**Supplemental Information**

**Supplemental Materials and Methods**

**Cell viability assay**

hAECs were seeded in 100 µl (1.25x10^5^ cells/cm^2^) in 96-well plates and cultured with serial concentrations of SB-431542 for 7 days. The medium was changed every 2 or 3 days. Cell viability was analyzed using a Cell Counting Kit-8 (CCK-8, Dojindo Laboratories, Japan) according to the manufacturer’s instructions. The absorbance was measured at 450 nm using a microplate reader (iMark Microplate Absorbance Reader, Bio-Rad Laboratories, CA, USA). After confirming the concentration of SB-431542, hAECs were cultured in the presence or absence of SB-431542. Cell viability was analyzed on day 1, day 3, and day 5 using the CCK-8 assay.

**Immunocytochemistry**

hAECs cultured on cover glasses were fixed for 15 min at room temperature (RT) with 4% formaldehyde/PBS. After washing with PBS, the cells were permeabilized with 0.3% Triton X-100 in PBST for 15 min. The samples were incubated with blocking buffer consisting of PBS supplemented with 3% bovine serum albumin and Tween 20 for 30 min. Subsequently, they were incubated with the appropriate primary antibody diluted in blocking buffer overnight and washed three times with PBS. After primary antibody incubation, the samples were incubated with the appropriate secondary antibody for 60 min at RT. The primary antibody was an anti-NANOG antibody (10 µg/ml, R&D Systems, MN, USA), and the secondary antibody was rabbit anti-goat IgG (H+L) (Alexa Fluor™ 488, 4 µg/ml, Thermo Fisher Scientific, MA, USA). Then, the samples were stained with Alexa Fluor™ 647-conjugated phalloidin (Thermo Fisher Scientific, MA, USA) for 20 minutes at room temperature and mounted with ProLong Glass Antifade Mountant with NucBlu™ for nuclear staining. Fluorescence images were captured under the same optical conditions using a confocal microscope (TCS SP8, Leica, Germany). To analyze the fluorescence intensity, the mean gray values in 30 regions of interest (ROIs) from three optical fields were measured in the green (NANOG) channel and analyzed using ImageJ Fiji (NIH, MD, USA). Each ROI was defined according to the location of DAPI-stained nuclei.

**Legends to Supplemental Figures**

**Figure S1.**

(A) RT–qPCR analysis of *CDH2* expression in hAECs cultured with serial concentrations of SB-431542. The data are shown as the mean ± SEM.

(B) Viability of hAECs cultured with serial concentrations of SB-431542.

(C) Proliferation of hAECs cultured in the presence or absence of SB-431542. The data are shown as the mean ± SEM (n = 3)

**Figure S2.**

(A) GSEA using the gene set associated with regionalization (GO: 0003002).

(B) GSEA using the gene sets of different germ layers: endoderm development (GO: 0007492), mesoderm development (GO: 0007498), and ectoderm development (GO: 0007398).

**Figure S3.**

(A) Comparison of immunocytochemical staining of NANOG (green) and F-actin (red) and DAPI staining (blue) between control and SB-treated cells on day 7. Scale bar: 50 μm.

(B) Comparison of the fluorescence staining intensity of NANOG between control and SB-treated cells on day 7. ***p < 0.001 (Wilcoxon rank-sum test).

**Supplemental Tables**

**Table S1. List of primer sequences for RT–qPCR**

| Primer name |  | Sequence |
| --- | --- | --- |
| *PPIA* | Forward | ATGCTGGACCCAACACAAAT |
|  | Reverse | TCTTTCACTTTGCCAAACACC |
| *CDH1* | Forward | TCGACACCCGATTCAAAGTG |
|  | Reverse | GTCCCAGGCGTAGACCAAGA |
| *CDH2* | Forward | CTCCTATGAGTGGAACAGGAACG |
|  | Reverse | TTGGATCAATGTCATAATCAAGTGCTGTA |
| *SNAIL* | Forward | TGCAGGACTCTAATCCAAGTTTACC |
|  | Reverse | GTGGGATGGCTGCCAGC |
| *ITM2A* | Forward | TCTCGTAGGCCTTTCCTTCA |
|  | Reverse | AGGCAGGAAGTAGGGCTCTC |
| *ELF3* | Forward | CATGACCTACGAGAAGCTGAGC |
|  | Reverse | GACTCTGGAGAACCTCTTCCTC |

**Table S2. List of genes used for GSEA**

| Gene set* | Gene symbol | | | | | | | | | | |
| --- | --- | --- | --- | --- | --- | --- | --- | --- | --- | --- | --- |
| Stemness-related | *DPPA4* | *ELOVL6* | *FGF2* | *GDF3* | *GJA1* | *HSPA4* | *KLF4* | *KLF5* | *LIN28A* | | *MYBL2* |
|  | *MYC* | *NANOG* | *NR2E1* | *POU5F1* | *PRDM14* | *SFRP2* | *SIX2* | *SOX2* | *TDGF1* | | *ZIC3* |
| Ectoderm development GO:0007398 | *AMER2* | *BMPR1A* | *EDA2R* | *EPB41L5* | *FZD7* | *GRHL3* | *ITGA6* | *ITGAM* | *KRT6B* | | *LHX1* |
|  | *MIR145* | *NF2* | *SHH* | *SMURF1* | *STX2* | *VAX2* | *VPS52* | *ZBTB17* | *ZBTB7B* | |  |
| Endoderm development GO:0007492 | *APELA* | *ARC* | *BMP4* | *BMPR1A* | *BPTF* | *CDC73* | *COL11A1* | *COL12A1* | *COL4A2* | | *COL5A1* |
|  | *COL5A2* | *COL6A1* | *COL7A1* | *COL8A1* | *CTNNB1* | *CTR9* | *DKK1* | *DUSP1* | *DUSP2* | | *DUSP4* |
|  | *DUSP5* | *EOMES* | *EPB41L5* | *EXT1* | *FGF8* | *FN1* | *GATA4* | *GATA6* | *GDF3* | | *HDAC1* |
|  | *HMGA2* | *HNF1B* | *HOXC11* | *HSBP1* | *INHBA* | *ITGA4* | *ITGA5* | *ITGA7* | *ITGAV* | | *ITGB2* |
|  | *ITGB5* | *KIF16B* | *LAMA3* | *LAMB1* | *LAMB3* | *LAMC1* | *LEO1* | *LHX1* | *MACROH2A1* | | *MED12* |
|  | *MESP1* | *MIXL1* | *MMP14* | *MMP15* | *MMP2* | *MMP8* | *MMP9* | *NANOG* | *NKX2-1* | | *NODAL* |
|  | *NOG* | *NOTCH1* | *ONECUT1* | *PAF1* | *PAX9* | *PELO* | *POU5F1* | *RTF1* | *SETD2* | | *SMAD2* |
|  | *SMAD3* | *SMAD4* | *SOX17* | *SOX2* | *SOX7* | *SSBP3* | *TBX20* | *VTN* | *ZFP36L1* | |  |
| Mesoderm development GO:0007498 | *ACVR1* | *ACVR2A* | *AMH* | *APELA* | *BMP4* | *BMP7* | *BMPR1A* | *BMPR2* | *BMX* | *BTK* | |
|  | *CER1* | *CITED2* | *CRB2* | *CTDNEP1* | *DAND5* | *DKK1* | *DLL3* | *EOMES* | *EPB41L5* | *EPHA2* | |
|  | *ETV2* | *EXOC4* | *EXT1* | *EXT2* | *EYA2* | *FGF8* | *FGFR2* | *FOXC1* | *FOXC2* | *FOXF1* | |
|  | *FOXH1* | *GDF11* | *GJA1* | *HAND1* | *HCK* | *HES7* | *HMGA2* | *HOXA11* | *IKZF1* | *IKZF3* | |
|  | *INHBA* | *IRX3* | *ITGA2* | *ITGA3* | *ITGA8* | *ITGB1* | *ITGB3* | *ITGB4* | *JAK2* | *KDM6B* | |
|  | *KLF4* | *LDB1* | *LEF1* | *LHX1* | *MESD* | *MESP1* | *MESP2* | *MEST* | *MIR1-1* | *MIR145* | |
|  | *MIR150* | *MIR200C* | *MIXL1* | *MSGN1* | *NF2* | *NODAL* | *NOG* | *NUP133* | *OSR1* | *OVOL1* | |
|  | *PALB2* | *PAX2* | *POFUT2* | *POGLUT1* | *POU4F1* | *PPP2CA* | *PRKACA* | *PRKAR1A* | *PUS7* | *RPL38* | |
|  | *RPS6KA6* | *SCX* | *SECTM1* | *SETD2* | *SFRP2* | *SHH* | *SIX2* | *SMAD1* | *SMAD2* | *SMAD3* | |
|  | *SMAD4* | *SMO* | *SNAI1* | *SRF* | *TAL1* | *TBX1* | *TBX19* | *TBX20* | *TBX3* | *TBX6* | |
|  | *TBXT* | *TCF15* | *TEAD2* | *TIE1* | *TLX2* | *TP63* | *TRIM15* | *TSPY1* | *TSPY10* | *TSPY2* | |
|  | *TSPY3* | *TSPY4* | *TSPY8* | *TWSG1* | *TXNRD1* | *VEGFA* | *WNT11* | *WNT3* | *WNT3A* | *WNT5A* | |
|  | *YAP1* | *ZFP36L1* | *ZFPM2* |  |  |  |  |  |  |  | |
| Regionalization GO: 0003002 | *ACVR1* | *ACVR2A* | *ACVR2B* | *ACVRL1* | *ADGRG1* | *AHI1* | *AIDA* | *ALDH1A2* | *ALX4* | *AR* | |
|  | *ARC* | *ARL13B* | *ASCL1* | *ATM* | *ATP6AP2* | *AURKA* | *AXIN2* | *BARX1* | *BASP1* | *BHLHE40* | |
|  | *BHLHE41* | *BMI1* | *BMP2* | *BMP4* | *BMPR1A* | *BMPR1B* | *BMPR2* | *BPTF* | *BTG2* | *C1QA* | |
|  | *C3* | *CDON* | *CDX1* | *CDX2* | *CDX4* | *CELSR2* | *CER1* | *CFC1* | *CFC1B* | *CHRD* | |
|  | *CHSY1* | *CITED2* | *COBL* | *CRB2* | *CRKL* | *CTNNBIP1* | *CXXC4* | *CYP26B1* | *CYP26C1* | *DBX1* | |
|  | *DCANP1* | *DDIT3* | *DKK1* | *DLL1* | *DLL3* | *DLL4* | *DLX1* | *DLX2* | *DMRT2* | *DMRT3* | |
|  | *DMRTA2* | *DNAAF1* | *DPCD* | *DSCAML1* | *DVL2* | *DYNC2H1* | *DZIP1 L* | *EDN1* | *EGR2* | *EMX1* | |
|  | *EMX2* | *EN1* | *EP300* | *EPB41L5* | *EXT1* | *FBXL15* | *FEZF1* | *FEZF2* | *FGF1* | *FGF10* | |
|  | *FGF8* | *FGFR2* | *FKBP8* | *FOXA1* | *FOXA2* | *FOXB1* | *FOXC1* | *FOXC2* | *FOXD1* | *FOXF1* | |
|  | *FOXG1* | *FOXH1* | *FOXJ1* | *FOXN4* | *FRS2* | *FUZ* | *FZD5* | *GATA4* | *GATA5* | *GBX2* | |
|  | *GDF11* | *GDNF* | *GLI1* | *GLI2* | *GLI3* | *GORAB* | *GPC3* | *GPR161* | *GREM1* | *GRSF1* | |
|  | *GSC* | *GSX2* | *HELT* | *HES1* | *HES2* | *HES3* | *HES4* | *HES5* | *HES6* | *HES7* | |
|  | *HEY1* | *HEY2* | *HEYL* | *HHEX* | *HHIP* | *HIPK1* | *HIPK2* | *HOXA10* | *HOXA11* | *HOXA2* | |
|  | *HOXA3* | *HOXA4* | *HOXA5* | *HOXA6* | *HOXA7* | *HOXA9* | *HOXB1* | *HOXB2* | *HOXB3* | *HOXB4* | |
|  | *HOXB5* | *HOXB6* | *HOXB7* | *HOXB8* | *HOXB9* | *HOXC10* | *HOXC13* | *HOXC4* | *HOXC5* | *HOXC6* | |
|  | *HOXC9* | *HOXD10* | *HOXD11* | *HOXD13* | *HOXD3* | *HOXD4* | *HOXD8* | *HOXD9* | *IFT140* | *IFT172* | |
|  | *IFT52* | *IFT57* | *INTU* | *IRX1* | *IRX2* | *IRX3* | *ISL1* | *ITGAM* | *KAT2A* | *KDM2B* | |
|  | *LDB1* | *LEF1* | *LFNG* | *LHX1* | *LHX3* | *LMX1B* | *LRP2* | *LRP4* | *LRP5* | *LRP5 L* | |
|  | *LRP6* | *MAFB* | *MDFI* | *MED12* | *MEF2C* | *MEGF8* | *MEOX1* | *MEOX2* | *MESP1* | *MESP2* | |
|  | *MIB1* | *MLLT3* | *MNS1* | *MSGN1* | *MSX1* | *MTF2* | *MYF5* | *MYF6* | *NBL1* | *NEUROD1* | |
|  | *NEUROG1* | *NKD1* | *NKX2-1* | *NKX2-2* | *NKX3-1* | *NLE1* | *NODAL* | *NOG* | *NOTCH1* | *NOTCH2* | |
|  | *NOTO* | *NR2F2* | *NRARP* | *NRP2* | *NTF4* | *OSR1* | *OTX1* | *OTX2* | *OVOL2* | *PALB2* | |
|  | *PAX1* | *PAX2* | *PAX8* | *PBX1* | *PBX2* | *PBX3* | *PCDH8* | *PCGF2* | *PCSK5* | *PGAP1* | |
|  | *PIFO* | *PITX2* | *PKD1L1* | *PLD6* | *PLXNA2* | *POFUT1* | *POGLUT1* | *PRKACA* | *PRKACB* | *PRKDC* | |
|  | *PSEN1* | *PTCH1* | *RARG* | *RBPJ* | *RELN* | *RFX4* | *RING1* | *RIPPLY1* | *RIPPLY2* | *RNF2* | |
|  | *ROBO1* | *ROBO2* | *ROR2* | *RPGRIP1 L* | *SCMH1* | *SEMA3A* | *SEMA3C* | *SEMA3F* | *SENP2* | *SETDB2* | |
|  | *SFRP1* | *SFRP2* | *SHH* | *SIX1* | *SIX2* | *SIX3* | *SKI* | *SMAD2* | *SMAD3* | *SMAD4* | |
|  | *SMAD6* | *SMARCD3* | *SMO* | *SNAI1* | *SOSTDC1* | *SOX1* | *SOX17* | *SP8* | *SPRY1* | *SRF* | |
|  | *SUFU* | *TASOR* | *TBC1D32* | *TBR1* | *TBX1* | *TBX18* | *TBX19* | *TBX20* | *TBX3* | *TBX6* | |
|  | *TBXT* | *TCAP* | *TCF15* | *TCTN1* | *TDGF1* | *TDGF1P3* | *TDRD5* | *TGFBR1* | *TIFAB* | *TMED2* | |
|  | *TMEM107* | *TP53* | *TP63* | *TRA2B* | *TSHZ1* | *TULP3* | *VAX2* | *WDR19* | *WDR77* | *WLS* | |
|  | *WNT1* | *WNT11* | *WNT2* | *WNT2B* | *WNT3* | *WNT3A* | *WNT5A* | *WNT7A* | *WNT7B* | *WNT8A* | |
|  | *WNT8B* | *WT1* | *XRCC2* | *YY1* | *ZBTB16* | *ZIC3* |  |  |  |  | |

***** Gene set obtained by Gene Set Enrichment Analysis (https://www.gsea-msigdb.org/gsea/index.jsp)
