# Supplementary figures and images for: Inhibition of Epithelial-Mesenchymal Transition Maintains Stemness in Human Amniotic Epithelial Cells
Source: Stem Cell Rev Rep. 2022 Aug 6;18(8):3083–91. doi: 10.1007/s12015-022-10420-1 (PMC9622541; doi:10.1007/s12015-022-10420-1)

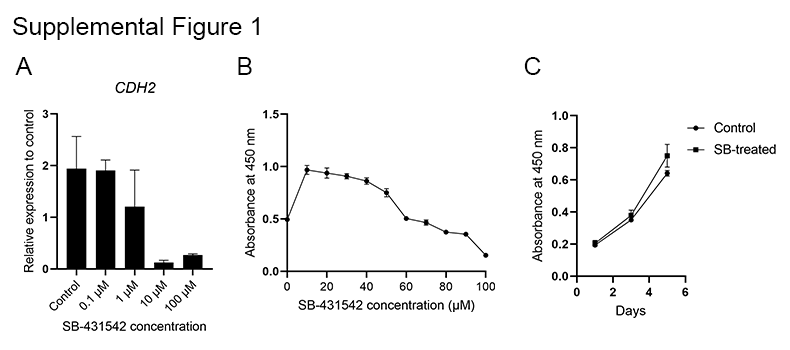

Supplement: Supplementary file 2 — (PNG 43 kb) [file 12015_2022_10420_Fig4_ESM.png]

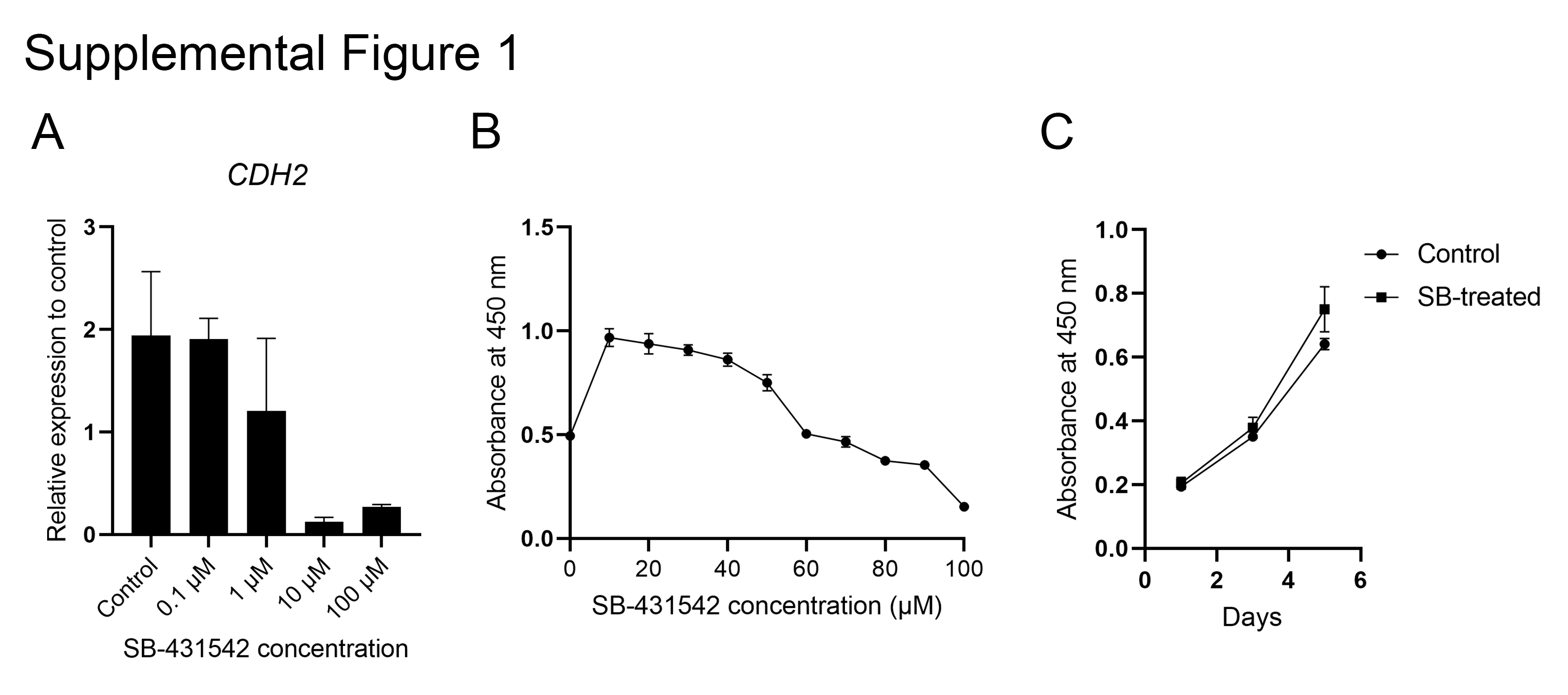

Supplement: Supplementary file 3 — High resolution image (TIF 13978 kb) [file 12015_2022_10420_MOESM2_ESM.tif]

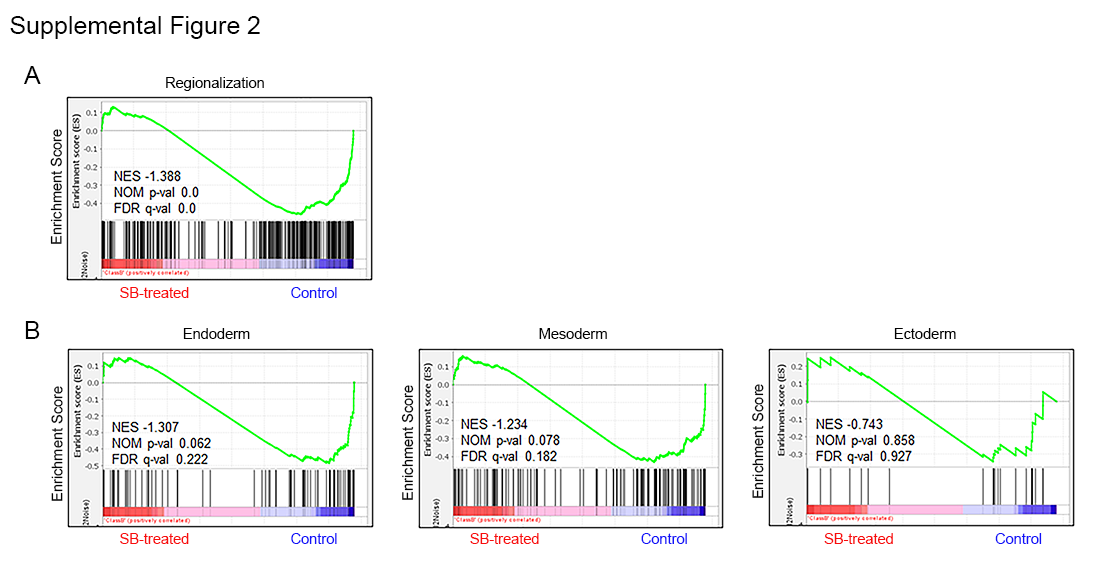

Supplement: Supplementary file 4 — (PNG 1302 kb) [file 12015_2022_10420_Fig5_ESM.png]

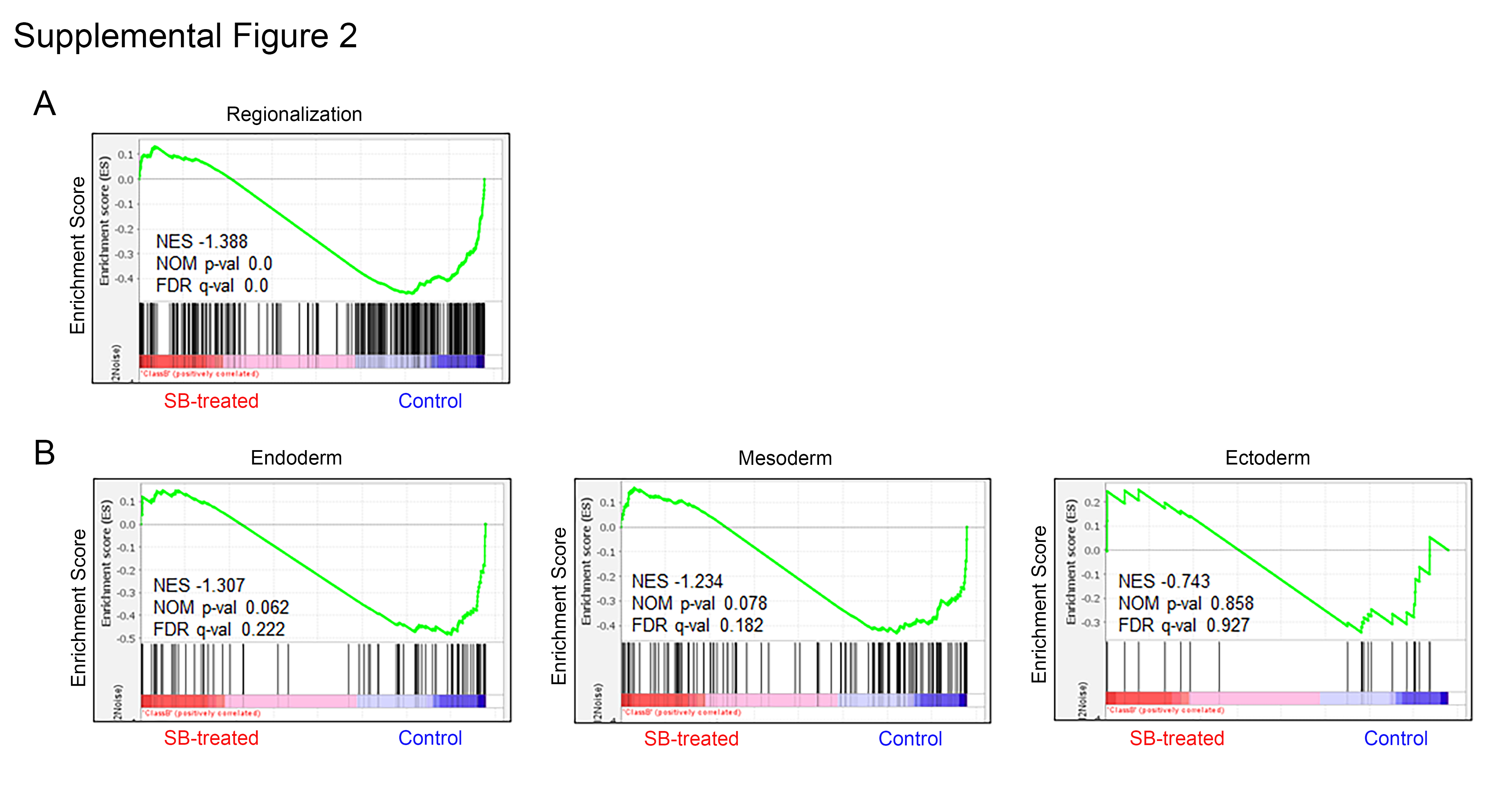

Supplement: Supplementary file 5 — High resolution image (TIF 35969 kb) [file 12015_2022_10420_MOESM3_ESM.tif]

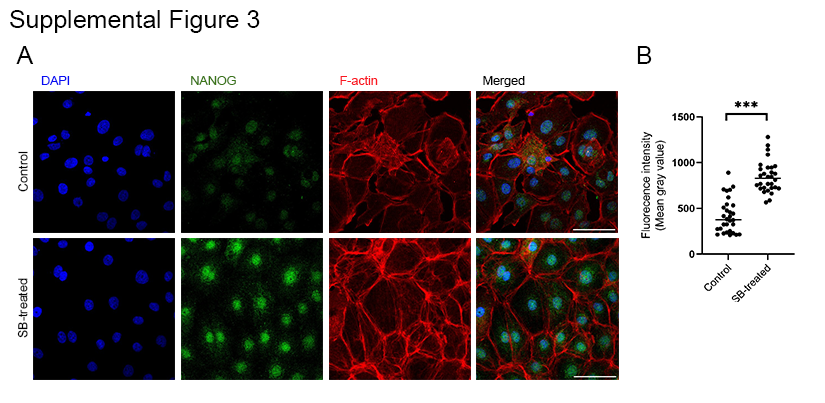

Supplement: Supplementary file 6 — (PNG 263 kb) [file 12015_2022_10420_Fig6_ESM.png]

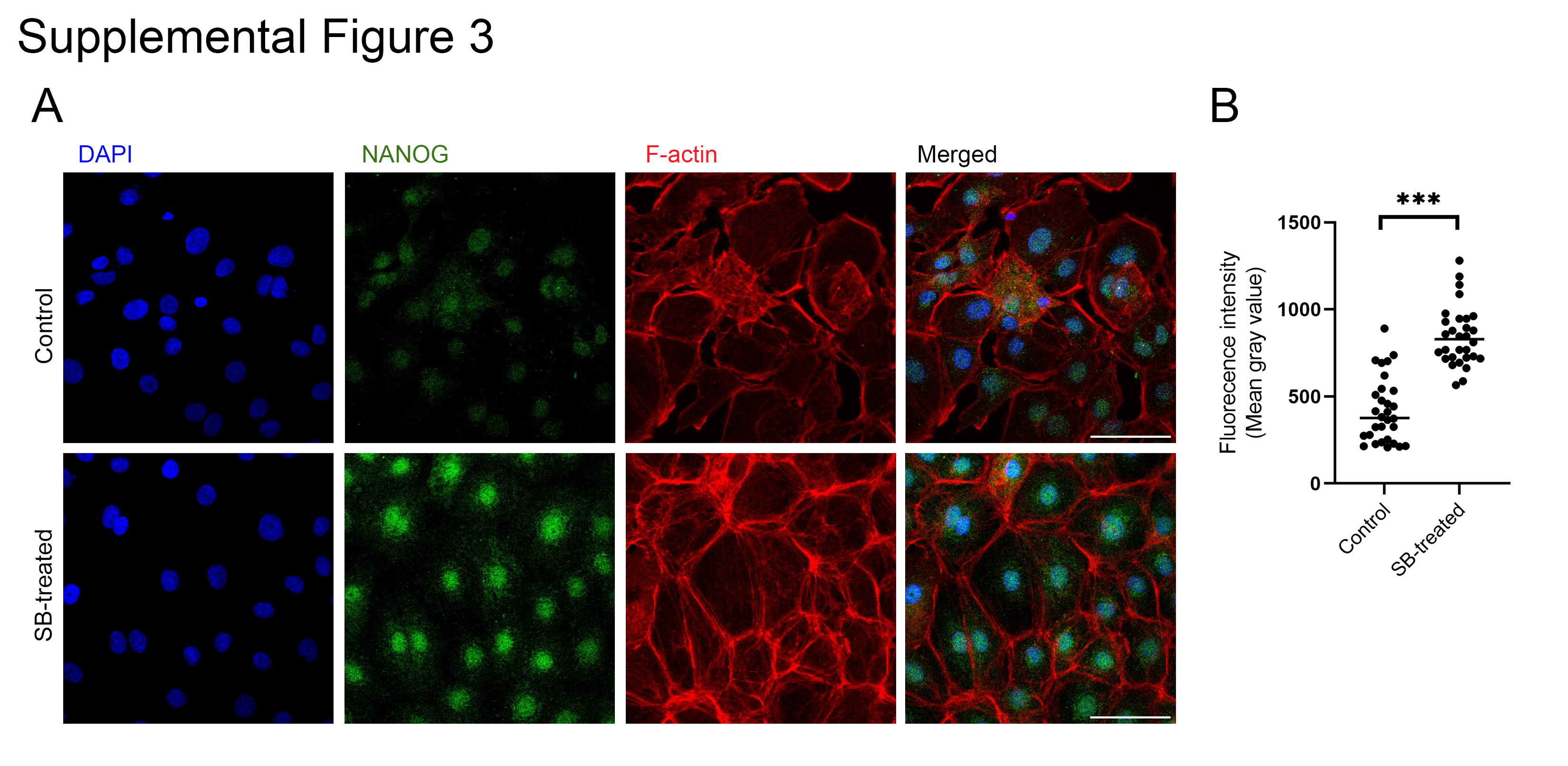

Supplement: Supplementary file 7 — High resolution image (TIF 25716 kb) [file 12015_2022_10420_MOESM4_ESM.tif]
